# Propranolol for familial cerebral cavernous malformation (Treat_CCM): study protocol for a randomized controlled pilot trial

**DOI:** 10.1186/s13063-020-4202-x

**Published:** 2020-05-12

**Authors:** Silvia Lanfranconi, Elisa Scola, Giulio Andrea Bertani, Barbara Zarino, Roberto Pallini, Giorgio d’Alessandris, Emanuela Mazzon, Silvia Marino, Maria Rita Carriero, Emma Scelzo, Giuseppe Faragò, Marco Castori, Carmela Fusco, Antonio Petracca, Leonardo d’Agruma, Laura Tassi, Piergiorgio d’Orio, Maria Grazia Lampugnani, Enrico Bjorn Nicolis, Antonella Vasamì, Deborah Novelli, Valter Torri, Jennifer Marie Theresia Anna Meessen, Rustam Al-Shahi Salman, Elisabetta Dejana, Roberto Latini, the Treat-CCM Investigators, R. Pallini, G. d’Alessandris, F. Pignotti, C. Sturiale, A. Albanese, S. Lanfranconi, E. Scola, G. A. Bertani, B. Zarino, G. Valcamonica, D. Ronchi, M. R. Carriero, E. Scelzo, G. Faragò, S. Pogliani, U. de Grazia, C. Bossi, E. Mazzon, S. Marino, R. Ciurleo, C. Fusco, A. Petracca, L. D’Agruma, P. Raggi, A. Simeone, P. d’Orio, M. G. Lampugnani, E. Dejana, E. B. Nicolis, A. Vasamì, D. Novelli, V. Torri, J. M. T. A. Meessen, R. Latini, G. Balconi, A. Foresta, M. G. Buratti, M. Carrara, M. L. Ojeda Fernandez, R. Al-Shahi Salman, R. Treglia, A. P. Maggioni, E. Beghi, M. Tettamanti, C. Regna-Gladin, A. Prelle, M. Mangiavacchi, Marco Poloni, F. Lazzaroni, M. Malinverno, D. Novelli, E. B. Nicolis, M. G. Buratti, A. Vasami, C. Ungaro, F. Raucci, M. Castori, L. Tassi

**Affiliations:** 1 Department of Neurology, Fondazione IRCCS Cà Granda Ospedale Maggiore Policlinico, Padiglione Monteggia—piano 3, Via Francesco Sforza 35, 20122 Milan, Italy; 2 Department of Neuroradiology, Fondazione IRCCS Cà Granda Ospedale Maggiore Policlinico, Via Francesco Sforza 35, 20122 Milan, Italy; 3 Department of Neurosurgery, Fondazione IRCCS Cà Granda Ospedale Maggiore Policlinico, Via Francesco Sforza 35, 20122 Milan, Italy; 4 Department of Neurosurgery, Università Cattolica del Sacro Cuore, Largo Francesco Vito 1, 00168 Rome, Italy; 5 IRCCS Centro Neurolesi “Bonino Pulejo”, Contrada Casazza, 98124 Messina, Italy; 6 Cerebrovascular Disease Unit, Fondazione IRCCS Istituto Neurologico Carlo Besta, Via Giovanni Celoria 11, 20133 Milan, Italy; 7 Department of Neuroradiology, Fondazione IRCCS Istituto Neurologico Carlo Besta, Via Giovanni Celoria 11, 20133 Milan, Italy; 8 Division of Medical Genetics, Fondazione IRCCS Casa Sollievo della Sofferenza, Viale Cappuccini 2, 71013 San Giovanni Rotondo, Italy; 9 “Claudio Munari” Epilepsy Surgery Centre, ASST Grande Ospedale Metropolitano Niguarda, Piazza dell’Ospedale Maggiore 3, 20162 Milan, Italy; 10 Laboratory of Vascular Biology, IFOM, Firc Institute for Molecular Oncology, Via Adamello 16, 20139 Milan, Italy; 11 Laboratory of Cardiovascular Clinical Pharmacology, Mario Negri Institute for Pharmacological Research-IRCCS, Via Mario Negri, 2, 20156 Milan, Italy; 12 Laboratory of Research Methodology, Mario Negri Institute for Pharmacological Research-IRCCS, Via Mario Negri, 2, 20156 Milan, Italy; 13 Centre for Clinical Brain Sciences, University of Edinburgh, Little France Crescent 49, Edinburgh, EH16 4SB UK

**Keywords:** Cerebral cavernous malformation, Propranolol, Magnetic resonance imaging

## Abstract

**Background:**

Cerebral cavernous malformations (CCMs) are vascular malformations characterized by clusters of enlarged leaky capillaries in the central nervous system. They may result in intracranial haemorrhage, epileptic seizure(s), or focal neurological deficits, and potentially lead to severe disability. Globally, CCMs represent the second most common intracranial vascular malformation in humans, and their familial form (FCCMs) accounts for one-fifth of cases. Neurosurgical excision, and perhaps stereotactic radiosurgery, is the only available therapeutic option. Case reports suggest that propranolol might modify disease progression.

**Methods:**

Treat_CCM is a prospective, randomized, open-label, blinded endpoint (PROBE), parallel-group trial involving six Italian clinical centres with central reading of brain magnetic resonance imaging (MRI) and adverse events. Patients with symptomatic FCCMs are randomized (2:1 ratio) either to propranolol (40–80 mg twice daily) in addition to standard care or to standard care alone (i.e. anti-epileptic drugs or headache treatments). The primary outcome is intracranial haemorrhage or focal neurological deficit attributable to CCMs. The secondary outcomes are MRI changes over time (i.e. de novo CCM lesions, CCM size and signal characteristics, iron deposition, and vascular leakage as assessed by quantitative susceptibility mapping and dynamic contrast enhanced permeability), disability, health-related quality of life, depression severity, and anxiety (SF-36, BDI-II, State-Trait Anxiety Inventory).

**Discussion:**

Treat_CCM will evaluate the safety and efficacy of propranolol for CCMs following promising case reports in a randomized controlled trial. The direction of effect on the primary outcome and the consistency of effects on the secondary outcomes (even if none of them yield statistically significant differences) of this external pilot study may lead to a larger sample size in a definitive phase 2 trial.

**Trial registration:**

ClinicalTrails.gov, NCT03589014. Retrospectively registered on 17 July 2018.

## Background and rationale

Cerebral cavernous malformations (CCMs) are vascular lesions consisting of clusters of abnormally dilated blood vessels (capillaries). These lesions are mainly located in the central nervous system (brain and spinal cord) and, more rarely, affect the skin and retina. CCMs have typical raspberry-like appearance due to their composition of multiple bubble-like structures called caverns. Each cavern is filled with blood and lined by a layer of endothelial cells. In the case of CCMs, the bubble-like caverns are grossly dilated vessels that leak due to defects in the endothelial cells and due to the loss of other structural components that are required for vessel wall integrity. Lesion size is variable, ranging from microscopic to a few inches in diameter. Subjects may be asymptomatic or present a wide variety of symptoms including seizures, intracranial haemorrhages, or focal neurological deficits [1–3].

The CCM prevalence is approximately one out of every 500–600 people. However, such an estimation strongly depends on the methodology of ascertainment. Autopsy studies indicate the prevalence of CCMs to be between 0.2% and 0.5% of the population, while papers using brain MRI clinical series report a prevalence between 0.39% and 0.9% [4–6].

CCMs occur in sporadic or familial (FCCM) forms. Multiple lesions are more common in FCCMs and the number of lesions is strongly correlated with the patient’s age. The diagnosis of FCCMs is established with the presence of either or both of the following: multiple CCMs, and multiple family members with one or more CCM. The identification of a heterozygous germline, pathogenic variant in *KRIT1*, *CCM2*, or *PDCD10* confirms the diagnosis of FCCMs [7]. Genetic screening is positive in 96% of families with multiple affected members, and in 57% of sporadic cases with multiple lesions (more so if new lesions form over time) [2]. While FCCM patients bear ubiquitous germline heterozygous mutation of CCM genes, the analysis of the surgically removed cavernomas discloses a second somatic mutation in the same CCM gene. This suggests that local genetic homozygosity is indispensable to prime the pathogenic cascade. The analysis of affected tissues often discloses a second somatic mutation, which is likely indispensable to prime the pathogenetic cascade, suggesting a two-hit mechanism [8–10]. The prevalence of FCCMs, highly variable among different populations and case series, ranges from 0.2 to 3/10,000 subjects [11, 12]. Multiple CCMs, suggesting FCCMs, affect roughly one-fifth of people with CCMs [1]. Nonetheless, these new findings on genetic determinants have not impacted on actual treatment of CCMs.

Although the presentation of CCMs is not uncommon in children, individuals often show the first sign of symptoms in their 20s or 30s. Globally, the annual bleeding rate is 2.5% per patient-year, with prior haemorrhage and brain stem location being the major risk factors for CCMs to bleed [1, 13].

CCM is an endothelial disease as demonstrated in murine models. In fact, only mice with endothelial-selective inducible ablation of FCCM genes develop cerebral vascular malformations with morphological and molecular features similar to humans [14–16]. The activity of the three members of the complex, encoded by the three FCCM-related genes, has to converge in the same pathway, since the morphology and specific brain localization of the vascular malformations is roughly comparable in the three genetic forms of FCCM.

No pharmacological treatment is at present available to inhibit the formation of new malformations, to stabilize the existing ones and stop their progression. To date, the standard of care is represented by treatment of CCM-associated clinical manifestations, such as headache and epilepsy, and consists of anti-epileptic drugs or drugs for recurrent headache [17]. The only available treatments are neurosurgical excision or stereotactic radiosurgery. Surgical removal of lesions associated with intractable seizures or focal deficits from recurrent haemorrhage or mass effect may be considered [13, 18, 19], with at times significant complications [20]. Neither neurosurgery nor radiosurgery can completely eradicate the multiple lesions of FCCMs, which increase in number over time. Thus, pharmacological therapies are urgently needed, particularly in FCCMs.

Propranolol has efficacy, documented in RCTs, for the treatment of infantile haemangiomas, another common vascular lesion affecting the skin [21–24]. To date, only anecdotal reports have been published supporting its efficacy in CCMs [19, 25–27]. In mice, propranolol was effective in preventing CCM expansion and in reducing vascular permeability (Matteo Malinverno et al., 2019, unpublished data).

The recognized safety of propranolol, as demonstrated by millions of patients of all ages, including children treated over the last 40 years [28], makes this drug a good candidate for a pragmatic, external, pilot randomized trial.

## Methods/design

### Aims

The purpose of this exploratory pilot trial is to test whether 2-year treatment with propranolol in addition to standard care can reduce the incidence of clinical events, as compared to standard care in patients with FCCMs. Furthermore, this trial will help to set up future clinical trials in an area where there is to date no approved drug reducing the progression of CCM lesions, and will pave the way to study the mechanisms underlying the possible therapeutic role of propranolol in CCMs.

### Design

**Fig. 1:**
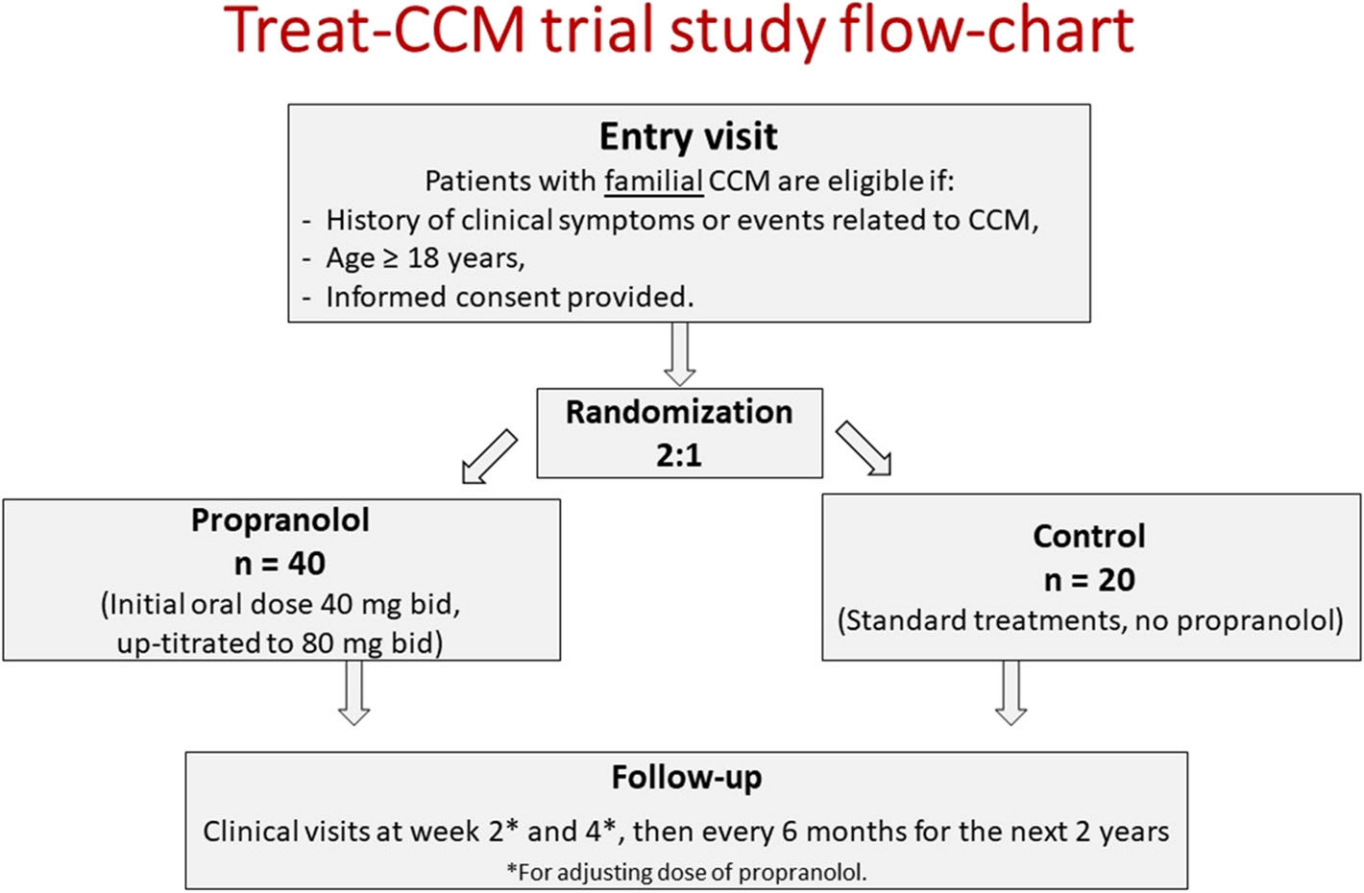
Study flow chart. bid twice daily, CCM cerebral cavernous malformation

The study protocol was designed according to the real-life management of patients with FCCMs (Fig. 1). Treat_CCM is a multicentre, open-label, randomized controlled trial (PROBE design, prospective randomized open trial with blinded evaluation of outcomes) in patients with FCCMs. Patients, fully informed of the trial, after signing the informed consent form are randomly assigned by computer, upon completing the randomization electronic case report form (e-CRF), in a 2:1 ratio to the study groups: one receiving *propranolol* in addition to standard care; the other, *controls*, receiving standard care alone (Fig. 1). The study will be open-label since the implementation of blinding would require placebo and ad hoc procedures with costs non-sustainable in a pilot study fully supported by public funding. In order to reduce possible biases, a PROBE design will be applied so that each MRI examination will be centrally read after de-identification, and all adverse clinical events will be centrally adjudicated. It should be pointed out that by no means will surgery, whenever indicated, be delayed and/or avoided because of study treatment allocation.

A two-step trial [29] is proposed as follows:

- Step 1: duration 2 years; 60 patients, 40 in propranolol group and 20 controls; if no difference between the study groups in 2-year incidence of adverse clinical events (e.g. intracerebral haemorrhage (ICH) or focal neurological deficits (FND)) is found and different MRI variables suggest improvement, the second phase is started, to assess long-term efficacy of propranolol.
- Step 2: single-arm study, all patients in the propranolol group; the drug will be declared effective in the case that the patients’ condition is improved or remains stable over the 2-year follow-up (e.g. years 3 and 4). The threshold of proportion of responses will be defined upon analysis of the 2-year follow-up data.

### Population

Eligible patients will be selected from the six participating clinical centres (Fondazione IRCCS Cà Granda Ospedale Maggiore Policlinico, Milan; Policlinico Universitario Agostino Gemelli, Rome; Fondazione IRCCS Istituto Neurologico Carlo Besta, Milan; Fondazione IRCCS Casa Sollievo della Sofferenza, San Giovanni Rotondo; IRCCS Centro Neurolesi “Bonino Pulejo”, Messina; ASST Grande Ospedale Metropolitano Niguarda Milan). In addition, other patients will be identified via CCM patient organizations, such as Associazione Nazionale Angioma Cavernoso Cerebrale ONLUS (https://www.anacc.net/). Inclusion and exclusion criteria are listed in Table 1.

**Table 1:** Inclusion and exclusion criteria

Inclusion criteria	
1	Patients with familial cerebral cavernous malformations (FCCM)
2	History of clinical symptoms or events: ICH, seizures, stroke, permanent or transient focal deficits, intellectual disability, or any other neurological symptoms supposedly related to CCM
3	Age ≥18 years
4	Written informed consent to participate in the study prior to any study procedures
Exclusion criteria	
1	Implanted pacemaker or any other condition preventing the MRI examination
2	Bradycardia (< 50 bpm), second or third-degree AV block or symptomatic hypotension
3	Unstable diabetes
4	Severe asthma
5	Liver and/or renal failure
6	Current use of verapamil or diltiazem for risk of excessive bradycardia
7	Previous brain surgery (within 6 months)
8	Known hypersensitivity to study drug (propranolol or any of the ingredients)
9	Pregnant or lactating women, or women at risk of childbearing who are not under protection by an accepted method of contraception
10	Participation to another clinical trial
11	Inability to cooperate with the trial procedures
In addition to these specific exclusion criteria, all well-documented contraindications to beta-blocker use are also valid in this trial	

*AV* atrioventricular, *CCM* cerebral cavernous malformation, *ICH* intracerebral haemorrhage, *MRI* magnetic resonance imaging

### Intervention

Propranolol is a non-selective beta-blocker, indicated for the treatment of hypertension and other cardiovascular and neurological disorders. The recommended initial oral dose for the group randomized to propranolol is 40 mg twice daily, as recommended in Italian Pharmacopeia, to be up-titrated to 80 mg twice daily in the absence of excessive bradycardia (e.g. heart rate < 50 bpm) or hypotension. However, doses as low as 10 mg twice daily and up to 160 mg twice daily, 20–320 mg daily, are acceptable according to the individual’s tolerance of side effects. Both study groups will receive recommended standard care. Study treatment will be continued for the whole duration of the study.

### Outcomes

The primary endpoint will be the new occurrence of clinical symptomatic CCM-related events, which are intracerebral haemorrhage (ICH) and focal neurological deficits (FND), as defined by Al-Shahi Salman et al. [30], excluding seizures.

Secondary endpoints include the following:

1. Microvascular haemorrhages as assessed by MRI analysis of brain tissue magnetic susceptibility, a biophysical property proportional to the local iron content (susceptibility weighted imaging (SWI) and quantitative susceptibility mapping (QSM)), and dynamic contrast enhanced permeability (DCEP).
2. Clinical outcomes, other than ICH and FND, such as global disability, health-related quality of life, depression severity, and two types of anxiety (state anxiety, or anxiety about an event, and trait anxiety, or anxiety personal level), as assessed by the SF-36, BDI-II and State-Trait Anxiety Inventory (STAI) questionnaires.
3. Seizures.
4. Different MRI CCM characteristics, such as location (cerebellum, brainstem, right/left hemispheric white matter, right/left basal ganglia), diameter, length, and MRI signal appearance. Lesions with previous surgical treatment will be excluded from imaging analysis.
5. Appearance of de novo CCM lesions at MRI.

### Exploratory targets

#### Brain imaging

MRI is, at present, the objective non-invasive tool to track in vivo the evolution of CCMs. Recently, in vivo MRI assessment of iron deposition and vascular permeability with advanced MRI techniques, such as QSM and DCEP, have been proposed as objective and quantifiable biomarkers of disease activity in CCMs with potential application in natural history and clinical trials [31], expanding conventional imaging in cerebral cavernous malformations [32]. Iron leakage and vascular permeability have a central role in CCM pathogenesis and their quantification can be an objective biomarker of disease activity in CCMs. Therefore, the quantification of biomarkers of DCEP and QSM with in vivo MRI and their longitudinal changes may correlate with the clinical behaviour of CCMs [33]. Patients will undergo brain MRI at randomization and at 1 and 2 years of follow-up.

#### Biobanking/biomarker study

The identification of circulating biomarkers in a population of CCM patients with recorded clinical history and documented therapeutic response will potentially contribute to establish prognostic and response-to-therapy indicators in the peripheral blood of CCM patients. Biomarkers to monitor CCM are urgently needed and are potentially fruitful according to several reports on other cerebrovascular disorders [34]. Indeed, specific circulating miRNAs have been reported in infantile haemangiomas which respond to propranolol [35], in cerebral aneurisms [36], and for cerebral haemorrhagic and ischaemic stroke [37]. Patients will undergo blood sampling for biobanking at randomization, at 1 month, and at 1 and 2 years of follow-up.

#### Microbiome

In a murine model of FCCMs, it has been reported that the microbiome composition influences the progression of brain angiomas [38]. Recent data in 75 patients go along the same line [39] and therefore it is of interest to characterize patients included in Treat_CCM at baseline as well as during disease evolution. Stools will be sampled for microbiome analysis at randomization, at 1 month, and at 1 and 2 years of follow-up.

### Follow-up phase

**Fig. 2:**
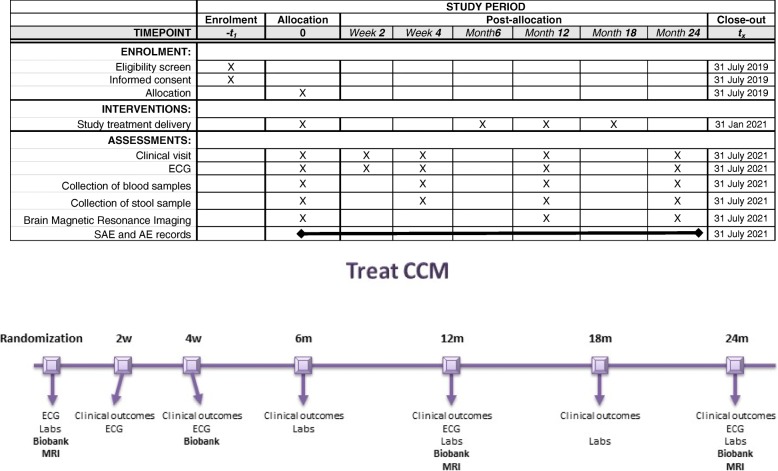
Study plan and timeline according to the Standard Protocol Items: Recommendations for Interventional Trials (SPIRIT) statement

Follow-up clinical visits will be performed at weeks 2 and 4 for dose adjustment of the study medication plus adverse events and side effect monitoring, and then every 6 months until the study end at month 24. Visits at 12 and 24 months are mandatory for MRI, and blood and faeces sampling for biobanking. Six-month and 18-month visits can be substituted with a telephone interview by a member of clinical study site staff to check for her/his vital status and whether any serious adverse event has occurred (Fig. 2).

### Sample size

To the best of the available knowledge, the 2-year risk of CCM-related events (ICH and FND) was estimated at 10.1% in CCM patients receiving recommended standard care [1]. Upon performing a formal sample size calculation, 834 patients should be included in the study to demonstrate a risk reduction of 50% with a power of 80% at a significance level of one-tailed α = 0.05. This estimate is unrealistic both for the over-optimistic treatment effect size and for the number of patients to be included, incompatible with a rare disease.

Therefore, in order to obtain preliminary data, a pilot study is planned, adopting a confidence interval approach, rather than using the more usual power and statistical significance method [40]. The sample size calculation for the pilot trial is driven by the proposed sample size of the main trial. According to the nature of the pilot study design, an 80% one-sided confidence interval is chosen, instead of the 95% two-sided interval usually considered for formal comparative trials. With 60 patients randomized in a 2:1 propranolol:control ratio, in the absence of a reduction of adverse events, a clinically meaningful effect, defined as ≥50% reduction of the 2-year risk of CCM-related events, can be excluded by the defined 80% upper confidence interval. Otherwise, the study will provide a promising signal of activity.

### Modality of data processing

The data and the samples collected for the study will be processed as coded data. The treating physician will identify each patient within the study with a code. This code will not enable direct identification, unless at the clinical centre where this code will be securely stored together with the patient’s name and surname. For biological analyses, researchers will identify samples and the data connected to them only through this code. No association between the results of scientific investigations on the samples and the patient’s identity will be possible.

De-identified biological samples will be stored, under UNI EN ISO 9001: 2015 regulations (for the following: collection, storage, and distribution of biological samples and related data for scientific research), transferred, and processed with modalities that guarantee their quality, integrity, availability, and traceability. All of the measures for the correct storage and handling will be put in place, according to the study protocol.

Only the treating physician, the monitors, and the regulatory authorities will have access to patient’s data at the clinical centre and will be able to associate the code with patient’s name for verification purposes. In particular situations, and only after the authorization of the clinical centre and of the promotor, Istituto di Ricerche Farmacologiche Mario Negri, its delegates may also have access to identifiable data.

### Quality control and trial monitoring

The safety profile of propranolol is reassuring and is documented by millions of patients of all ages, including infants treated mostly, but not only, for cardiovascular disorders over the last 40 years. Despite the expected very low probability of adverse effects related to propranolol, patients shall be controlled in particular for heart rate, blood pressure, and other adverse reactions possibly attributable to propranolol.

Quality control activities will be applied by the Study Secretariat at IRCCS-Mario Negri (IRFMN) to each stage of data handling to ensure data are recorded and reported in compliance with the protocol. All of the electronic case report forms (e-CRFs) will be reviewed for completeness and accuracy at the Study Secretariat by trained staff; errors and omissions will be entered on data query forms returned to the investigator for resolution. The Data Safety and Monitoring Board (DSMB) will monitor the safety data in the project in an ongoing basis. Serious and non-serious adverse events (SAEs and AEs) that coincide with primary and secondary endpoints will be collected on the e-CRFs and evaluated by the DSMB. Investigators shall report to the Study Secretariat (IRFMN) all SAEs suspected to be related to the study medications or any serious adverse drug reaction (SADR) within 24 h of learning of its occurrence.

On-site monitoring visits will be performed at site opening and at regular intervals throughout the study. On average, four on-site monitoring visits are foreseen for each participating site. During the visit, a certified monitor will verify adherence to the protocol: completeness, accuracy, and consistency of the data; and adherence to Good Clinical Practice (GCP) and local regulations on the conduct of clinical research. The investigator should guarantee access to source documents for the monitor. The clinical monitor will check participant medical records and other trial-related records (source documents) to verify the data reported to the sponsor on the e-CRFs and in all required reports. The clinical monitor will communicate deviations from the protocol to the investigators and will ensure that appropriate action designed to prevent recurrence of the detected deviations is taken and documented. Deviations from study protocol will be entered into the Protocol Deviation Log and communicated to the sponsor. All of the monitoring activities will be documented according to GCP rules and reported to the sponsor.

#### Study committees

In order to assess primary and secondary endpoints of this open trial, a PROBE design will be adopted. The following procedures will be implemented:

- Clearly defined objective outcomes: the primary outcome events of the study are based on objective clinical assessment, and the imaging secondary endpoint will be calculated from MRI examinations read in a central laboratory by experienced neuro-radiologists, unaware of patient identification and study treatment.
- Blinded Endpoint Adjudication Committee: all outcome events will be adjudicated by blinded adjudication experts. Blinding of all event documentation will be performed by trained personnel at the Study Secretariat. The role of the independent Clinical Event Committee will be to oversee the blinded adjudication of all primary events. This Committee will report to the Steering Committee. Records of all adjudication decisions and of Clinical Event Committee minutes will be maintained.
- Data handling: at the Study Secretariat, identification of treatment allocation on the e-CRF will only occur where necessary in order to minimize access to treatment information during data handling.
- Steering Committee: the Steering Committee will include one representative for each participating clinical centre, the Principal Investigator (ED, IFOM, sponsor of the trial), one representative of the Patient’s Association, and a representative responsible for the Study Secretariat and has the full responsibility for the planning, conduction, analysis, publication of the study protocol, and results.
- Clinical Event Committee (CEC): CEC members will be independent and will not have direct contact with patients randomized into this trial. The main roles and responsibilities of the CEC are: to agree on definitions of the clinical endpoints and on standard procedures for assessing these endpoints, and to validate blindly the events recorded and reported by the Investigators as endpoints of the study. A Clinical Event Committee manual will be prepared and approved by the members of the CEC.
- Data and Safety Monitoring Board (DSMB): the roles and responsibilities of the Data and Safety Monitoring Board (DSMB) will be defined by the same DSMB with special focus on intensive monitoring of the safety aspects in the whole study population. Safety reports will be made available to the DSMB by the study statistician every 6 months. No specific interim analysis is foreseen for efficacy, unless required by the Steering Committee or DSMB because of safety concern.

#### Centralized laboratories

- MRI Core Laboratory (Policlinico MI): examinations will be sent as digital recordings to the MRI Core Laboratory, where a central reading will be performed. A users’ manual for MRI will be made available to the participating centres to ensure common standard methods of image acquisition.
- Biobank (IRFMN): the coordination of the collection, long-term storage of biological samples in a centralized UNI EN ISO 9001: 2015-certified (for the following: collection, storage, and distribution of biological samples and related data for scientific research) biobank, and distribution of de-identified biological samples. In particular, blood samples will be collected from patients at the time of MRI (randomization, 12 months, and 24 months) and at the 4-week visit.
- Circulating biomarker assays laboratory (IFOM): serum/plasma samples from patients before the beginning of the treatment will be used to characterize the circulating miRNAs and identify a CCM patient-specific miRNA signature in comparison to age-matched and sex-matched healthy individuals. In addition, we will test the power of the miRNA signature to monitor the response to the pharmacological treatment with propranolol and the progression of the disease [35, 41].
- Microbioma will be assessed by the 16S technique in stool samples collected at randomization, 12 months, and 24 months [38].

#### Guidelines for stopping treatment

A permanent discontinuation of trial medication should be considered only when one of the following conditions exists:

- A patient decides that it is in her/his best interest to withdraw her/his consent to continue study treatment
- A serious adverse event occurs that is suspected to be related to trial medication and/or prevents patient’s continuation on study medication
- An investigator considers it advisable for sound, explicit, and documented clinical reasons

In all of these cases, the scheduled follow-up visits will be continued as planned by the study protocol. A patient will be considered discontinued from the study only if he or she withdraws their consent to be followed by the participating centre, or he or she is lost to follow-up after exhausting all means of contact. Surgical correction of CCM during follow-up shall require the patient’s discontinuation from the study.

In the case of discontinuation, the status of the patient at the last visit or last available contact will be used for the final analysis. Vital status may be ascertained through public records, in the case of failure of all other methods of contact.

#### Statistical analyses

The main analysis will be performed according to an intention-to-treat (ITT) approach. Therefore, all patients randomized in the study will be included in the analysis. As we cannot conduct inferential statistics on the data collected in the pilot study, we will only compare the proportions of adverse events between the groups to assess the safety of propranolol [42]. A per-protocol analysis is foreseen.

Baseline characteristics will be presented by treatment groups, and descriptive statistics will be performed to assess the success of randomization. Kaplan–Meier curves will be constructed to illustrate the occurrence of adverse events in the study over time.

Concerning the analysis of this pilot study, we are only interested in whether the treatment estimate is larger or smaller than zero. Consequently, it is not necessary to formally undertake a hypothesis test of the results. An extension of the trial in the case of encouraging preliminary results is foreseen. Special care will be paid to the biologic consistency of the different endpoints, even if none of them will yield statistically significant differences.

## Discussion

To the best of our knowledge, from a search of ClinicalTrials.gov on 29 April 2019, at present only one interventional trial with long-term drug treatment of CCMs is registered, testing atorvastatin in 80 patients with CCMs (AT CASH EPOC; NCT02603328). This single-centre trial is double-blinded vs placebo, with 1:1 randomization [43].

Considering the lack of effective pharmacological treatments of CCMs, and the safety and wide availability of propranolol, there is a risk that this drug will be prescribed off-label also in the absence of clear evidence of benefit. Therefore, it is imperative to maximize inclusion and perform randomized trials as soon as possible.

Some limitations are to be acknowledged. Firstly, the non-blind design may cause biases in the reporting of subjective variables such as quality of life; however, the PROBE design should protect from major influences on endpoint variables. Secondly, the number of patients to be enrolled is suitable for a pilot trial, which may at best reassure the safety of the study drug in patients with CCMs and provide the rationale for a future adequately sized trial, difficult in view of the low prevalence of CCMs and possible non-evidence-based preferences for treatments [44].

Treat_CCM aims at triggering effective transnational collaboration in order to assess the efficacy of propranolol in FCCMs by extending access to the trial also to children. The efficacy of propranolol in the much more frequent sporadic form of the disease could also be tested. Moreover, in the case of encouraging results, as judged by the Steering Committee, a larger phase 2, single-arm trial may be started.

## Trial status

The first patient was included in Treat_CCM in March 2018, and, as of the end of April 2019, 57 patients have been included. Recruitment is ongoing and, according to the study protocol, each patient will be followed up for at least 2 years. The current protocol is version 2.5 created on 30 November 2018. Any protocol modifications will be communicated to relevant parties (e.g. trial participants) and published on relevant channels (e.g. Clinicaltrials.gov). The results of this trial will be published in an appropriate scientific journal.

## Data Availability

The datasets used and/or analysed during the current study are available from the corresponding author on reasonable request.

## Abbreviations

AE: Non-serious adverse event
CCM: Cerebral cavernous malformation
CEC: Clinical Event Committee
DCEP: Dynamic contrast enhanced permeability
DSMB: Data Safety and Monitoring Board
e-CRF: Electronic case report form
FCCM: Familial form of cerebral cavernous malformation
FND: Focal neurological deficits
GCP: Good Clinical Practice
ICH: Intracerebral haemorrhage
IRFMN: Istituto di Ricerce Farmacologiche IRCCS-Mario Negri
ITT: Intention to treat
MRI: Magnetic resonance imaging
PROBE: Prospective, randomized, open-label, blinded endpoint
QSM: Quantitative susceptibility mapping
SADR: Serious adverse drug reaction
SAE: Serious adverse event
SWI: Susceptibility weighted imaging
